# Combining Farmers’ Preferences With Evidence-Based Strategies to Prevent and Lower Farmers’ Distress: Co-design and Acceptability Testing of ifarmwell

**DOI:** 10.2196/27631

**Published:** 2022-01-11

**Authors:** Kate M Gunn, Gemma Skaczkowski, James Dollman, Andrew D Vincent, Camille E Short, Susan Brumby, Alison Barrett, Nathan Harrison, Deborah Turnbull

**Affiliations:** 1 Department of Rural Health Allied Health and Human Performance University of South Australia Adelaide Australia; 2 Freemason’s Centre for Male Health and Wellbeing The University of Adelaide Adelaide Australia; 3 Alliance for Research in Exercise, Nutrition and Activity Allied Health and Human Performance University of South Australia Adelaide Australia; 4 Melbourne Centre for Behaviour Change Melbourne School of Psychological Sciences and Melbourne School of Health Sciences University of Melbourne Melbourne Australia; 5 National Centre for Farmer Health Western District Health Service Hamilton Australia; 6 School of Medicine Deakin University Melbourne Australia; 7 School of Psychology The University of Adelaide Adelaide Australia

**Keywords:** farm, agriculture, rural, drought, mental health, stress, coping, online intervention, acceptance and commitment therapy

## Abstract

**Background:**

Farming is physically and psychologically hazardous. Farmers face many barriers to help seeking from traditional physical and mental health services; however, improved internet access now provides promising avenues for offering support.

**Objective:**

This study aims to co-design with farmers the content and functionality of a website that helps them adopt transferable coping strategies and test its acceptability in the broader farming population.

**Methods:**

Research evidence and expert opinions were synthesized to inform key design principles. A total of 18 farmers detailed what they would like from this type of website. Intervention logic and relevant evidence-based strategies were mapped. Website content was drafted and reviewed by 2 independent mental health professionals. A total of 9 farmers provided detailed qualitative feedback on the face validity of the draft content. Subsequently, 9 farmers provided feedback on the website prototype. Following amendments and internal prototype testing and optimization, prototype usability (ie, completion rate) was examined with 157 registered website users who were (105/157, 66.9%) female, aged 21-73 years; 95.5% (149/156) residing in inner regional to very remote Australia, and 68.2% (107/157) “sheep, cattle and/or grain farmers.” Acceptability was examined with a subset of 114 users who rated at least module 1. Interviews with 108 farmers who did not complete all 5 modules helped determine why, and detailed interviews were conducted with 18 purposively sampled users. Updates were then made according to adaptive trial design methodology.

**Results:**

This systematic co-design process resulted in a web-based resource based on acceptance and commitment therapy and designed to overcome barriers to engagement with traditional mental health and well-being strategies—ifarmwell. It was considered an accessible and confidential source of practical and relevant farmer-focused self-help strategies. These strategies were delivered via 5 interactive modules that include written, drawn, and audio- and video-based psychoeducation and exercises, as well as farming-related jokes, metaphors, examples, and imagery. Module 1 included distress screening and information on how to speak to general practitioners about mental health–related concerns (including a personalized conversation script). Modules were completed fortnightly. SMS text messages offered personalized support and reminders. Qualitative interviews and star ratings demonstrated high module acceptability (average 4.06/5 rating) and suggested that additional reminders, higher quality audio recordings, and shorter modules would be useful. Approximately 37.1% (52/140) of users who started module 1 completed all modules, with *too busy* or *not got to it yet* being the main reason for non-completion, and previous module acceptability not predicting subsequent module completion.

**Conclusions:**

Sequential integration of research evidence, expert knowledge, and farmers’ preferences in the co-design process allowed for the development of a self-help intervention that focused on important intervention targets and was acceptable to this difficult-to-engage group.

**Trial Registration:**

Australian New Zealand Clinical Trials Registry ACTRN12617000506392; https://www.anzctr.org.au/Trial/Registration/TrialReview.aspx?id=372526

## Introduction

### Background

Farming is an occupation that involves numerous physical and psychological hazards. In recent years, Australian farmers have faced increased exposure to natural disasters, particularly prolonged droughts, fires and floods [1]. Farmers often both live and work on their farms, with family members across multiple generations being involved, consequently blurring the line between work, home, and family roles, which adds to their stress [2-5]. Financial pressure, loss of control, and uncertainty about the future are also associated with environmental stressors and are thought to significantly increase the risk of farmers experiencing mental health problems [3,6,7]. The inability to control these stressors and the sense of hopelessness and entrapment they can engender are thought to be potential risk factors for rural male suicide [8]. Indeed, studies have found a significantly higher incidence of suicide among rural and remote populations compared with metropolitan populations [9,10] and between agricultural workers compared with other employed rural people [11,12].

At the same time, farmers are known to face numerous barriers to help seeking from traditional physical and mental health services. These barriers are structural, such as the limited availability of medical and psychological professionals [13], and attitudinal [14,15]. For generations, Australian farmers have been characterized as being independent, stoic, and skilled at solving practical problems [2,16]. However, in the context of help seeking for the management of psychological distress, traits such as stoicism, independence, and a strong desire to keep personal matters private, may in fact be maladaptive [17]. Recent Australian research has found that farmers were half as likely to have sought help from a general practitioner (GP) or mental health professional in the previous 6 months compared with other employed rural people [13].

Fortunately, the National Broadband Network has now been rolled out in Australia, increasing rural access to internet sites and services [18]. A recent survey of 2000 businesses within the Australian agricultural sector found that up to 95% now have access to the internet [19], and the use of the internet to access health services is known to be increasing in the rural population [20,21].

The delivery of evidence-based interventions on the web offers opportunities to overcome some traditional barriers to help seeking faced by these populations. There is emerging evidence that computerized cognitive behaviour therapy (CCBT) interventions are acceptable in rural communities [22], and an unpublished example of a CCBT intervention designed to address anxiety, depression, and social functioning in Scottish farmers is *Living Life to the Full* (although it reported limited success) [23]. Given farmers’ numerous barriers to help seeking and the strong perception within the industry that outsiders (including health professionals [24]) fail to understand their needs and way of life, the development of such interventions needs to be done carefully. Consumer involvement in intervention design ensures that interventions are relevant, usable, and culturally appropriate for the target audience [25,26], which in turn can improve intervention success [27].

### Objective

The purpose of this paper is to describe the co-design of content and functionality of a website that aims to help farmers adopt transferable coping strategies that are likely to help them effectively cope with stress. The second purpose of this research is to test the acceptability and feasibility of this website in a broader Australian farming population. The development of this website involved the sequential integration of research evidence, expert knowledge, and farmers’ preferences. Methodological guidance and examples such as the studies by O’Brien et al [28] and Short et al [29] and the work outlined in this paper, provide a transparent account of intervention co-design and development upon which other clinicians and researchers can build.

## Methods

### Overview

**Figure 1:**
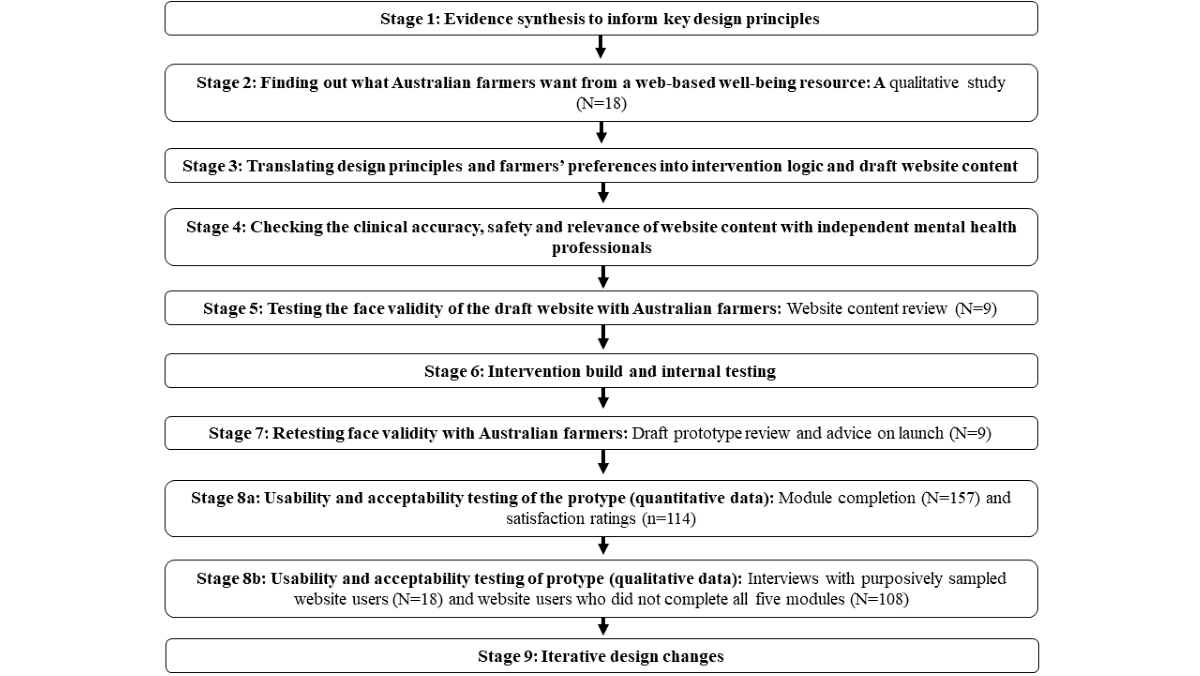
Development process for ifarmwell website.

Ethics approval for this project was granted by the University of South Australia human research ethics committee (application ID 0000035637). A 9-stage co-design process that included the sequential validation and optimization of evidence and expert opinion with farmers’ wants and preferences was used in a process similar to that described by Easton et al [30]. Each stage resulted in outputs (described in the *Results* section) that were used to inform the next stage of development. Figure 1 summarizes these stages.

In total, four key methodological approaches informed these stages: (1) synthesis of evidence from prior research to understand the problem and possible solutions (stages 1 and 2); (2) intervention mapping techniques to chart the logic of the intervention (including key acceptance and commitment therapy [ACT] processes or performance objectives, determinants of change, relevant behavior change strategies, and persuasive system design elements; stage 3) [31-33]; (3) a person-based approach via the involvement of farmers as co-designers [34,35] (stages 2, 5, and 7); and (4) iterative updating based on user feedback that allows for ongoing improvements to be made to the website (stages 8 and 9), which is informed by adaptive trial design methodology [36].

All farmers who participated in the research were adults who owned or played an active role in the operation of a farming or pastoral enterprise in Australia (or the spouse of someone who did), were fluent in English, had access to the internet, and had access to a mobile phone with reliable connection or reception at least once per week. The following 9-stage iterative process was conducted over a 3-year period.

### Stage 1: Evidence Synthesis to Inform Key Design Principles

Key learnings from published works [6,14,37-40], our own unpublished work, and views from relevant experts across the agricultural, financial, and mental health fields were summarized by the research team. The research team was well-placed to prioritize learnings, given their extensive knowledge of agriculture (KMG, SB, JD, and AB), behavior change interventions (DT, CES, and KMG), web-based interventions (KG, CES, and SB), and rural health (KG, SB, JD, AB, and NH) and mental health (KMG and DT).

### Stage 2: Finding Out What Australian Farmers Want From a Web-Based Well-being Resource—A Qualitative Study

#### Participants

A total of 11 male (11/18, 61%) and 7 female farmers (7/18, 39%), who met the above criteria, participated in the interviews. They had a median age of 45.5 years and were all from grain, sheep, and/or cattle farms across 4 Australian states.

#### Procedures

As described in detail elsewhere [41], participants were recruited via articles in print, radio and web-based media, advertising via relevant rural organizations, and personal and professional contacts of the research team. Telephone interviews were used to explore the farmers’ current internet use practices and preferences for websites designed to promote their mental health and well-being. Thematic analysis was used to analyze the verbatim interview transcripts [42]. Data were arranged under each theme in a Microsoft Excel spreadsheet using a framework approach. The data were checked for any evidence of themes that contradicted the key design principles identified in stage 1.

### Stage 3: Translating Design Principles and Farmers’ Preferences Into the Intervention Logic and Draft Website Content

The logic of the intervention was systematically developed by KG to ensure that important intervention targets (identified in stage 1 and explained further in the *Results* section) were addressed and that the effectiveness of the targets could be systematically assessed later. This included mapping the module content to the core ACT processes (acceptance, cognitive defusion, being present, self as context, values, and committed action [43]). It also included ensuring that relevant behavior change techniques [31] (outlined in the *Results* section) were included throughout to help address each of the behavioral determinants (ie, knowledge, skills, emotion, action planning, beliefs about capabilities, beliefs about consequences, motivation and goals, and memory, attention, or decision-making processes) thought to influence whether a user would successfully adopt the core ACT processes. The selection of these behavior change techniques was based on what has been previously shown to effectively address relevant behavioral determinants [31]. Although some overlap with behavior change techniques and persuasive system design elements is acknowledged, persuasive system design elements (as defined by Kelders et al [33] and outlined in the *Results* section) were also built into the intervention logic to help maximize user engagement and limit dropout.

The text, video, and audio content contained within each website module were then drafted by KG by integrating the key design principles from stage 1, farmers’ preferences established in stage 2, and the intervention logic identified in stage 3. Her first-hand experience of using ACT in her role as a clinical psychologist, living on a farm in a farming family, developing self-help mental health materials for rural populations, and formal training in intervention mapping, assisted with this process. The general principles of adult learning [44] were also considered.

### Stage 4: Checking the Clinical Accuracy and Safety of Website Content With Independent Mental Health Professionals

#### Participants

A male social worker with a long history of supporting drought-affected farmers and knowledge of and experience using ACT clinically and a female clinical psychologist highly experienced with clinical and forensic mental health populations and in the use of ACT, participated in this stage of testing.

#### Procedures

Independent feedback on the clinical accuracy, safety, and relevance of website content was provided on all website content using tracked changes in a Microsoft Word processing document. Suggestions were then incorporated where feasible (ie, would not make the modules too long) to enhance clinical impact.

### Stage 5: Testing the Face Validity of the Draft Website With Australian Farmers—Website Content Review

#### Participants

A total of 9 farmers (4/9, 44% men and 5/9, 56% women), who met the criteria outlined above and had participated in stage 2, took part in this stage of the research (herein referred to as *co-designers*). They ranged in age from 34 to 62 years and were from grain, sheep or cattle properties in the states of South Australia (7/9, 78%) and Western Australia (2/9, 22%).

#### Procedures

A copy of the draft website content was sent to the co-designers via post or email. Participants were also asked to comment specifically on several logo and design options (colors, fonts, background images, and layouts) provided as PDF files. Interviews were then conducted over the phone (or, in one case, in person) to gather feedback, with a focus on language, relevance, and face validity.

#### Analysis

Where possible, key recommendations for improvement were compiled, and edits were made to the working draft document following the completion of each interview.

### Stage 6: Intervention Build and Internal Testing

The purpose of this stage was to produce a working intervention prototype. The research team supplied the website content and design documents developed in earlier steps to a web developer and then worked in close collaboration with them to ensure that lessons from previous stages were integrated into the website and technical glitches were addressed. The prototype was made public in February 2018.

### Stage 7: Retesting the Face Validity of the Draft Website With Australian Farmers—Website Prototype Review and Advice on Launch

#### Participants

A total of 4 farmers (co-designers; 1/4, 25% male and 3/4, 75% female) provided detailed feedback on the website prototype. They were aged 24, 40, 61, and 62 years and were from grain, sheep, and/or cattle properties in South Australia (2/4, 50%), Western Australia (1/4, 25%), and New South Wales (1/4, 25%). A further 5 farmer co-designers (all men) provided feedback specifically on the website launch. They were aged 34, 44, 47, 53, and 55 years and were from sheep or cattle properties (1/5, 20%) or grain, sheep and/or cattle properties (4/5, 80%) in South Australia.

#### Procedures

Co-designers were sent a link to the website prototype along with broad instructions to work through the website and provide email or phone comments on any aspects they thought required changing.

#### Analysis

Key recommendations from participant comments were compiled and implemented where possible.

### Stage 8a: Usability and Acceptability Testing of the Prototype (Quantitative Data)

The trial was registered with the Australian New Zealand Clinical Trials Registry (ACTRN12617000506392) on April 3, 2017.

#### Participants

Usability testing of the prototype was conducted by 157 farmers who registered during the study period and met the criteria outlined above. Acceptability testing was undertaken with a subset of 114 users who provided a rating out of 5 for at least module 1. Their demographic characteristics are shown in Table 1.

**Table 1:** Demographics for all eligible registered users and those users who provided acceptability ratings for at least one module (stage 8a).

Characteristics			All registered users (N=157)		Users who provided acceptability ratings (N=114)
**Age (years)**					
	Values, mean (SD)	45.55 (12.17)		45.46 (12.65)	
	Values, median (range)	46 (21-73)		46 (21-73)	
**Gender, n (%)**					
	Female	105 (66.9)		79 (69.3)	
	Male	52 (33.1)		35 (30.7)	
**Remoteness of residence, n (%)^a^**					
	Major cities of Australia	7 (4.5)		6 (5.3)	
	Inner regional Australia	66 (42.3)		46 (40.7)	
	Outer regional Australia	59 (37.8)		43 (38.1)	
	Remote Australia	16 (10.3)		13 (11.5)	
	Very remote Australia	8 (5.1)		5 (4.4)	
**Farm type, n (%)**					
	Dairy	19 (12.1)		15 (13.2)	
	Grain, sheep and/or cattle	63 (40.1)		43 (37.7)	
	Horticulture, market garden, or fruit	14 (8.9)		9 (7.9)	
	Poultry	3 (1.9)		2 (1.8)	
	Sheep and/or cattle	44 (28)		37 (32.5)	
	Viticulture	1 (0.6)		1 (0.9)	
	Other	13 (8.3)		7 (6.1)	
**Education level (highest qualification), n (%)**					
	Postgraduate degree	17 (10.8)		13 (11.4)	
	University degree or diploma	70 (44.6)		49 (43)	
	Trade certificate	43 (27.4)		34 (29.8)	
	Finished high school	25 (15.9)		17 (14.9)	
	Finished primary school	2 (1.3)		1 (0.9)	
Hours per week spent using the internet, mean (SD)			16.42 (10.47)^b^		16.07 (10.23)

^a^n=156 and n=113 because of missing data.

^b^n=155 because of missing data.

#### Procedures

Consent for participation was established when users registered with the website. Data were collected from all users who registered between February and October 2018 inclusive.

#### Analysis

Analyses were conducted using SPSS Statistics for Windows (version 26; IBM Corp) [45]. Usability and acceptability were captured in several ways.

##### Star Ratings (Out of 5) by Each User at the Completion of a Module

At the end of each module, users were asked to rate that module on a scale ranging from 1 to 5 *stars*, where 1=unhelpful, 2=neutral, 3=satisfactory, 4=helpful, and 5=very helpful. The star rating out of 5 was used as it allowed for the multifaceted nature of acceptability to be captured [46] and because of the familiarity and briefness of this approach [47]. Acceptability ratings were examined for modules completed between February and October 2018. Ratings of acceptability for each module were estimated through a linear mixed model with maximum likelihood estimation, and the module number was entered as a fixed effect with 5 levels and a random intercept per participant. Baseline age, gender, education, farm type, remoteness, hours of internet use, psychological distress, and stress were also entered as fixed factors. The average acceptability rating for each user was calculated from the star ratings of all modules that a user completed.

##### Module Completion Rate

Data on module completion were captured beyond the February to October 2018 time frame (up to February 2020) to capture participants’ full record of participation (even if this was post-October 2018).

##### Association of Module Completion and Acceptability With Participant Demographics, Recent Exposure to Stressors and Distress Levels

During the registration process, demographics (gender, age, education level, and farm type), distress (Kessler Psychological Distress Scale [48]), and a single-item measure of exposure to stressors were completed. For the latter, users were asked to think of the most stressful situation they had encountered during the past month and rate how stressful they found this situation on a scale of 1 to 10 [40]. Residential postcodes were used to calculate remoteness using the Accessibility and Remoteness Index of Australia from the Australian Bureau of Statistics [49]. Owing to small numbers, the categories *finished high school* and *finished primary school* were combined for analysis. Similarly, poultry farming and viticulture were grouped with *other* farm type.

The association between demographics, stress exposure, distress, and module acceptability was examined using the mixed model described above. A series of univariable and multivariable linear regressions examined the relationship between the number of modules completed and demographic or distress and stress variables. Finally, Pearson correlations were used to examine the association between module completion and an individual’s average acceptability rating and the rating of the last module they completed.

### Stage 8b: Usability and Acceptability Testing of the Prototype (Qualitative Data)

#### Participants and Procedures

##### Brief Phone Calls With Users Who Did Not Complete All 5 Modules (to Find Out Why)

A total of 108 website users who had not continued with the next module within 5 weeks of completing the previous module were followed up with 2 phone calls, 1 email, and 1 additional attempt via email or phone approximately 1 month after that. Successful follow-ups were used to determine the reasons for not continuing with the modules so that we could find ways to enhance the website and aid engagement. Verbatim notes were taken during the phone calls along with email responses, which were manually analyzed by AB and KG using conventional content analysis [50] and a Microsoft Excel spreadsheet. Categories were derived from the data and reworked until all the data could be accounted for. Discrepancies between the coders were rare but were worked through until full agreement was reached.

##### Detailed Phone Interviews With Purposively Sampled Group of Users

A total of 18 farmers (7/18, 39% men and 11/18, 61% women) who had used the website were purposively selected from website users to gain a variety of impressions (based upon state, farm type, average module acceptability score, gender, and age) and invited via email to take part in a telephone interview to share their experiences. Farmers ranged in age from 23 to 71 years and were from dairy (1/18, 6%), horticulture (2/18, 11%), viticulture (1/18, 6%), sheep and/or cattle properties (7/18, 39%), and grain, sheep and/or cattle properties (6/18, 33%) in Victoria (6/18, 33%), New South Wales (4/18, 22%), South Australia (2/18, 11%), Tasmania (2/18, 11%), Western Australia (2/18, 11%), and Queensland (1/18, 6%). Interviews were audio recorded, transcribed verbatim, and analyzed by AB and KG using thematic analysis [42], with data arranged in a Microsoft Excel spreadsheet using a framework approach, and any discrepancies in coding discussed and reworked until full agreement was reached.

### Stage 9: Iterative Design Changes

Following the acceptability assessment of the prototype outlined above, the website was adapted to improve user experience. This aligns with the adaptive trial design methodology [36] and the person-based approach to intervention design of Yardley [34] by continuing to incorporate user feedback after live testing of the intervention.

## Results

### Stage 1: Evidence Synthesis to Inform Key Design Principles

A summary of our evidence synthesis and the key overarching design principles identified from this are shown in Table 2. In brief, farmers face many barriers to accessing traditional face-to-face mental health services, including a lack of service availability, cost, time, and concerns about confidentiality. They also perceive that outsiders (including health professionals) often do not understand the issues they face. The types of challenges that cause farmers the most stress are those that are beyond their control, and these are the things they feel least equipped to cope effectively with. However, *acceptance* has been shown to be an adaptive coping strategy for farmers in this context [40]. Together, these factors suggest that a new web-based mental health and well-being resource could help overcome existing barriers to engagement by being an accessible, confidential source of farmer-focused, practical self-help strategies based on ACT [51] if co-designed with farmers.

**Table 2:** Design principles resulting from the evidence synthesis.

Evidence synthesis	Resulting design principle
Barriers to accessing face-to-face mental health and well-being services in rural areas include cost, time, stigma, a lack of anonymity in country towns, a general lack of understanding of mental health issues, and the lack of availability of services [5,16,52-58].	Web-based resources may help to address barriers to the access and availability of services.
Barriers to help seeking for mental health issues among farmers include the desire for control, self-reliance, tendency to minimize the problem, and resignation [14,37]. Farmers prefer anonymous self-help books or internet resources [59].	Self-help resources align with farmers’ desire for control, self-reliance, and anonymity.
Farmers are often isolated and perceive a lack of understanding of rural issues from *outsiders* [6,38]. Many farmers report difficulty understanding health care professionals [14] and that health care professionals do not understand them and their way of life [6,53]. However, there is a high level of community trust within rural Australia [39], suggesting that a resource designed by farmers and for farmers may be considered credible.	Having a clear farming focus and co-designing alongside farmers is needed to ensure relevance and acceptability.
Managing uncertainty is a key challenge resulting from drought and a stressor that many farmers do not feel equipped to manage [6]. They are generally already good at solving problems, so they are less likely to benefit from assistance with that.	Uncertainty about the future is a key stressor that farmers need help with managing.
Information provision and educational resources alone are not enough to change key behaviors and thought processes [60]. Evidence-based behavior change techniques (eg, modeling, self-monitoring, and goal setting) should be built into web-based interventions to maximize the effect [33,61].	An interactive, engaging resource is needed.
Farmers who adopt acceptance as a coping strategy and do not engage in behavioral disengagement (giving up) are less likely to experience distress when faced with significant stressors during drought [40].	Acceptance is an effective coping strategy for farmers in this context.
ACT^a^ is a transdiagnostic, evidence-based psychotherapeutic approach that can foster acceptance and committed action (opposite of giving up) and improve well-being in a nonpathologizing way [62]. ACT may be used to address a range of psychological disorders and promote general well-being in nonclinical samples [62-64], including via web-based interventions [64,65]. It is particularly suited to contexts where the stressor must be accepted or cannot be fixed [66].	ACT may be an appropriate therapeutic model for this context.
Strategies to improve intervention adherence and effectiveness must also be included (eg, tunneling, personalization, and reminders) [33,67-69].	Issues relating to web-based intervention adherence need to be addressed.

^a^ACT: acceptance and commitment therapy.

### Stage 2: Finding Out What Australian Farmers Want From Web-Based Well-being Resources—a Qualitative Study

As reported elsewhere [41], farmers said that they would like a web-based resource that is easy to navigate and compatible with multiple devices and internet connections, as well as their sporadic internet use around work schedules. They preferred a casual and friendly tone, minimal use of jargon, and the inclusion of humor, and they requested information on when and how to seek additional professional help. They also said that they wanted a resource that was authentic, that reflected their challenges and way of life, and that they could see the benefits from quickly. There was no evidence of themes that contradicted the key design principles identified in stage 1.

### Stage 3: Translating Design Principles and Farmers’ Preferences Into the Intervention Logic and Draft Website Content

#### Overview

The resulting ifarmwell web-based intervention is a free, farmer-focused, password-protected self-help resource that contains 5 modules. Textbox 1 outlines the purpose of each module as explained to users, and Table 3 details the intervention logic and design, including the key content, targeted ACT processes, behavior change techniques, and persuasive system design elements contained within each module. The content is written for a low reading age (Gunning Fog score=5.8, easily understood by individuals aged 13-14 years) using friendly language with appropriate humor and farming-related metaphors and examples and fits with farmers’ ethos of independence and determination to help themselves. The intervention is nonpathologizing and focuses on improving *well-being* and *preventing* poor mental health rather than *treating* poor mental health or mental illness. The word *mental health* is avoided where possible on the website based on farmers’ advice about how best to engage their peers.

**ifarmwell module aims (as presented to users).**

**Module 1:** **Taking stock of your current well-being and some practical strategies to get you started**

- Confidentially discover how your current well-being compares with the well-being of other Australians
- Learn about additional support services that may be useful for you in addition to this web-based resource
- Provide some practical strategies tailored to specific challenges you may face

**Module 2:** **Thoughts are like bullies—how to spend less time *in your head***

- Understand the power thoughts have over the way you feel
- Become more aware of the thoughts or *stories* your mind plays to you
- Learn how to look at your thoughts rather than from them
- Practice evaluating whether a particular thought is helpful to tune in to or not
- Start to learn how to let go of unhelpful thoughts and focus on things that make life better

**Module 3: Doing what really matters—how to get the most out of life**

- Work out what is important to you
- Identify areas of life in which it would be useful to put more energy
- Recognize areas of your life in which it might be useful to put less energy

**Module 4: Training your *attention muscle* and focusing on the *here and now*—a more pleasant, less exhausting place to be**

- Become more aware of where your attention is and how this affects how you feel and behave
- Practice shifting attention to the here and now

**Module 5: Putting it all together and moving forward**

- Revisit strategies
- Plan out how to build these new strategies into day-to-day life
- Think about situations where familiar thoughts or stories may be triggered
- Plan how to respond to new challenges

**Table 3:** ifarmwell intervention logic and design.

ACT^a^ processes		Behavior change techniques (targeting key behavioral determinants of adoption of ACT processes)^b^	Persuasive system design elements (to aid engagement)^c^	Content details
**Module 1: taking stock of your current well-being and some practical strategies to get you started**				
	No ACT processes targeted	Self-monitoring Persuasive communication Information regarding outcomes Personalized messages Modeling or demonstration Goal setting or homework	Reduction Tunneling Tailoring Personalization Self-monitoring Praise Reminders Suggestion Similarity Liking Social learning Normative influence	Feedback from K10^d^ (current levels of distress) and COPE^e^ (current coping strategies) Personalized script for discussion with GP^f^ (if medium or high level of distress identified) Video demonstration of farmer speaking to GP about mental health using a script Psychoeducation tip sheets for 3 user-identified challenges Basic self-care and helpful coping strategies (default) Improving the quality of your sleep Managing conflict with others Improving the quality of your relationship How to get your point across Managing anger Coping with grief and loss Alcohol and drug use Dealing with domestic violence Adapting to new roles What to do if you are feeling down or low Coping after a natural disaster Succession planning Feeling trapped in an unhappy relationship What to expect in upcoming modules (intro to ACT) Homework planning or goal setting to implement tip sheet strategies
**Module 2:** **thoughts are like bullies—how to spend less time *in your head***				
	Acceptance Cognitive defusion Being present Self as context (being aware of your experiences without being attached to them)	Personalized messages Information regarding outcomes Self-monitoring Rewards or positive feedback (encouragement or reinforcement) Problem-solving Persuasive communication Prompts, triggers, and cues Rehearsal of relevant skills Graded tasks Goal setting or homework	Reduction Tunneling Tailoring Personalization Self-monitoring Praise Reminders Suggestion Similarity Liking Social learning Rehearsal	Homework review or problem-solving obstacles Feedback from Automatic Thoughts Questionnaire (identification of key challenging stories) Exploration of existing strategies tried to manage challenging stories. Worked? Pink sheep or elephants exercise; creative hopelessness Video: piece-of-paper metaphor demonstration Audio: notice thoughts while breathing (tool 1) Examining whether particular thoughts are helpful to focus on or not (drag and drop task with feedback; tool 2) Drafting thoughts in to just do it, plan a time, and let it go pens “I’m having the thought that...” exercise (tool 3) Giving stories a name exercise (tool 4) Identifying thinking errors (tool 5) Additional strategies to help you think differently about your thoughts (extra metaphors; tool 6) Homework planning or goal setting to implement strategies
**Module 3: Doing what really matters—how to get the most out of life**				
	Values Committed action	As detailed in module 2 above	Reduction Tunneling Tailoring Personalization Self-monitoring Praise Reminders Suggestion Similarity Liking	Homework review or problem-solving obstacles Consideration of current influences on behavior Valuing questionnaire and tailored feedback (removed, stage 9) Values clarification (drag and drop task) Reflection on current and future decision-making and interactions with others and considering values (tool 7) Planning to live more consistently with top 10 values in next week and next 6 months (acknowledge what already doing, schedule time, and plan to overcome obstacles; tool 8) Homework planning or goal setting to implement strategies
**Module 4:** **training your *attention muscle* and focusing on the *here and now*—a more pleasant, less exhausting place to be**				
	Being present Acceptance Cognitive defusion Self as context Values Committed action	As detailed in module 2 above, plus stress management, relaxation, or mindfulness	Reduction Tunneling Tailoring Personalization Self-monitoring Praise Reminders Suggestion Similarity Liking Rehearsal	Homework review or problem-solving obstacles Identifying existing activities fully present Audio: here and now exercise (tool 9) The basic (mindfulness) formula (tool 10) Audio: 5 slow, deep breaths grounding technique (tool 11) Audio: notice 3 things grounding technique (tool 12) Paying attention to 1 thing at a time when doing everyday activities (tool 13) Audio: letting go of difficult emotions (tool 14) Homework planning or goal setting to implement strategies
**Module 5: putting it all together and moving forward**				
	Acceptance Cognitive defusion Being present Self as context Values Committed action	As detailed in module 4 above	Reduction Tunneling Tailoring Personalization Self-monitoring Praise Reminders Suggestion Similarity Liking Social learning Rehearsal	Homework review or problem-solving obstacles Audio: leaves on a stream metaphor (tool 15) Video: normalizes difficulty in mastering these strategies and encourages persistence Summary of strategies (tool 16) Audio: cows on a truck metaphor (tool 17) Relapse prevention (warning signs): how to get yourself back on track and who you could turn to for extra help

^a^ACT: acceptance and commitment therapy.

^b^As defined by Michie et al [31].

^c^As defined by Kelders et al [33].

^d^K10: Kessler Psychological Distress Scale.

^e^The COPE inventory [70].

^f^GP: general practitioner.

**Figure 2:**
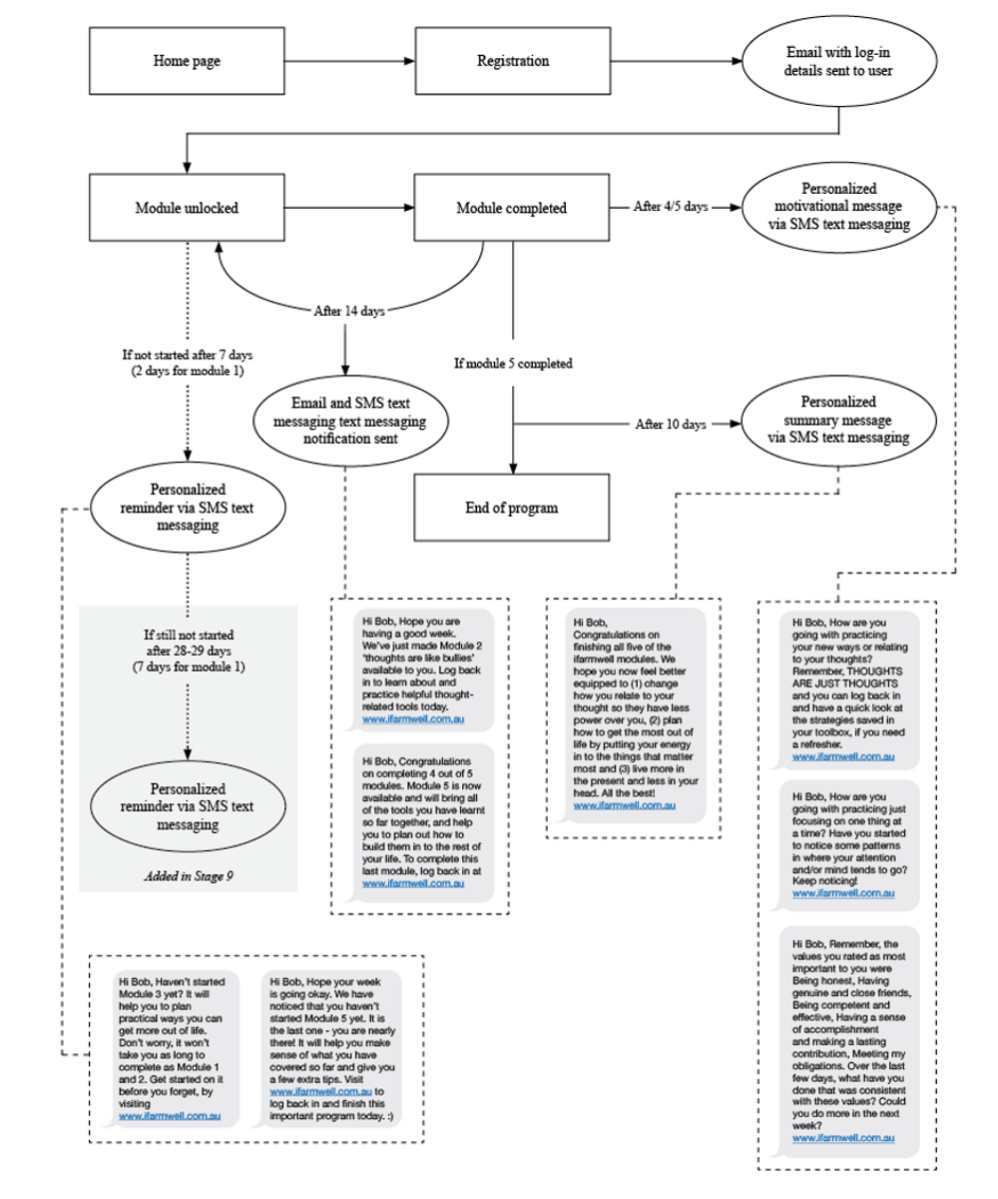
Wireframe of the ifarmwell website.

The intervention was completed over 10 weeks, with each module taking approximately 30 minutes. Users could access the intervention at any time and on any device with an internet connection (eg, laptop, desktop, tablet, and mobile phone). As shown in Figure 2, each module must be completed for the next module to be unlocked. This provided users with time to implement the strategies they learned from the previous module before moving to the next. This design was based on the literature showing that tunneled web-based interventions are less likely to overwhelm users and are better placed to personalize the intervention, leading to greater behavior change [67,68]. Figure 2 also indicates the frequency and type of SMS text messaging reminders sent to users throughout the intervention. Figure 2 shows the final design following changes made after the acceptability testing of the prototype (described in Stage 7).

#### Personalization

Tailored content was delivered throughout the intervention based on user responses and demographic variables. This included personalized imagery reflective of participants’ farming type, which has also been successfully used in farmer suicide stigma research [69]. In module 1, users were asked to complete the Kessler Psychological Distress Scale measure of distress and were provided with feedback about their current levels of distress, how these compared with others’ scores, and inform them if their scores suggested that they should seek professional face-to-face help. More specifically, based on their distress score, users were advised whether they were experiencing what was considered a low (10-19), mild (20-24), moderate (25-29), or severe (30-50) level of distress [71] and subsequently, whether it was recommended that they see their GP to discuss their well-being. Users were given the option of printing off the results of their web-based assessment and a script to guide a conversation about their mental health with their GP. Users were also presented with a short video showing someone else having that conversation with their GP. Finally, any severely distressed users (defined by cut-off) were contacted by a member of the research team by phone or email to encourage them to see a GP and remind them of helpline numbers.

At the end of each module, key tools were summarized, and users could choose to save them to their Toolbox if they found them useful. Modules 2 to 5 contained a homework review component, which asked users about the things they chose to focus on in the previous module and how much they had practiced them since. This section also asked users to reflect on whether anything *got in the way* or made doing this difficult and what they could do in the next week to overcome these difficulties.

At the end of the intervention, the Toolbox provided a summary of the user’s existing coping strategies, the stories the user’s mind often plays to them, their new preferred tools, and their top values (to guide future decision-making).

#### Module Content

Module content was transdiagnostic and useful for people experiencing a range of problems or conditions and for people simply wanting to improve their well-being or get more out of life. More specifically, module 1 was designed to take stock of users’ current well-being, suggest other sources of help if required, address basic self-care, and provide practical coping strategies that are targeted at users’ pressing, unique needs. This was based on a brief suite of questions used to identify the top 3 areas of need for each user. They were then presented with corresponding evidence-based tip sheets (eg, on sleep).

The remaining modules each focused on a particular ACT process. Module 2 addressed the power of thoughts and explained that avoidance or attempts to control difficult thoughts and feelings could be counterproductive. The module asked users to list the emotions and thoughts that they were struggling with, name the stories that they tell themselves, classify them as helpful to focus on, and identify errors in their thinking. Module 3 helped users clarify their values and find ways to live more consistently with them. Module 4 involved several mindfulness-based exercises (not labeled *mindfulness*-based upon farmers’ advice), designed to help users identify where their attention was and how this influenced them and practice shifting their attention to the *here and now*. Module 5 summarized the key strategies learned, examined possible triggers and warning signs to be aware of in the future, and reminded users of key sources of support.

### Stage 4: Checking the Clinical Accuracy and Safety of the Website Content With Independent Mental Health Professionals

Mental health professionals provided guidance on the appropriateness and safety of the content and suggested minor changes. These included grammatical edits, alterations to simplify the language (eg, *being clear about your values* changed to *knowing what matters to you*), and adding a few more detailed explanations and metaphors to explain key concepts (eg, your mind as an *ideas generator*). Additional reflective questions were also suggested, for example, “what happened to the thought?” following an exercise to help let go of distressing thoughts. It was also recommended that additional text be added to help normalize the fact that one’s ability to focus and shift attention may vary from day to day.

### Stage 5: Testing the Face Validity of the Draft Website With Australian Farmers—Website Content Review

Overall, participants felt that the module content was acceptable and relevant to farmers. Changes made to the content included repeating icons throughout the modules to guide the user, the inclusion of a summary of content at the beginning of each module, the inclusion of additional cartoons, and the removal of some references to *stress*, which farmers felt their peers would find off-putting (eg, *under pressure* rather than *stressed*). Additional methods for tailoring the content to farmers were also identified. For example, a co-designer suggested likening sorting out thoughts into different categories, to drafting sheep into different pens.

### Stage 6: Intervention Build and Internal Testing

A web-based intervention prototype that could be tested by users was created, and wireframes to summarize the website’s structure were developed, as detailed in Figure 2. Internal testing by members of the research team resulted in comprehensive lists of hundreds of technical revisions that needed to be made by web developers to improve the user experience.

### Stage 7: Retesting the Face Validity of the Draft Website With Australian Farmers—Website Prototype Review and Advice on Launch

This stage resulted in several changes to the look and *feel* of the website, such as a change of font color to improve readability and the inclusion of additional banner photographs featuring machinery and images of younger farmers to ensure broad appeal. Suggestions for improvement also included some website usability issues, such as the ease of saving and returning to a module later. Guidance was also provided on when would be a suitable time of year to advertise and launch the website (ie, not in January when many Australian grain farmers are on holidays after busy harvests in the lead up to Christmas). The website was made public in February 2018.

### Stage 8a: Usability and Acceptability Testing of Prototype (Quantitative Data)

#### Module Completion Rate

A total of 157 users (described in Table 4) registered on the website between February 2018 and October 2018 and were eligible to participate in the study. Of the 157 users, 17 (10.8%) users registered but did not start module 1. Table 4 shows the total number of people starting and completing each module.

The completion rates for modules 1 to 5 among those that commenced each module were 83.6% (117/140), 89% (81/91), 94% (68/72), 100% (58/58), and 100% (52/52), respectively. Approximately 35% (49/140) of the people who started module 1 did not start module 2 (*dropout*). The dropout rates for modules 2 to 4 were 21% (19/91), 19% (14/72), and 10% (6/58), respectively. Overall, 37.1% (52/140) of the people who started module 1 completed the entire intervention. The median time between starting module 1 and starting module 5 was 16 weeks (8 weeks was intended if users had 2 weeks break before commencing the next module), with a range of 8 to 76 weeks (15/52, 29% took 8-12 weeks; 28/52, 54% took 13-24 weeks; and 9/52, 17% took >24 weeks).

**Table 4:** Number of users starting and completing each module.

	Started module	Completed module	Completion rate (%)
Module 1	140	117	83.6
Module 2	91	81	89
Module 3	72	68	94.4
Module 4	58	58	100
Module 5	52	52	100

#### Star Ratings (Out of 5) Provided by Each User at the Completion of a Module

A total of 310 acceptability star ratings were submitted by 114 unique users (those who completed at least module 1 before October 2018). Of 114 users, the average rating across all modules on a 1- to 5-star rating scale was 4.06 (SD 0.99), with 17 (14.9%) people providing an average rating of 1 to 3, 43 (37.7%) people providing an average rating of >3 to 4, and 54 (47.4%) people providing an average rating of >4. The adjusted acceptability ratings for each module from the linear mixed model are shown in Table 5, and the mixed model is shown in Multimedia Appendix 1. There was a significant difference between module ratings; the module 3 acceptability rating was significantly lower than modules 1 (
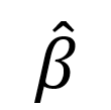
=0.52, 95% CI 0.28-0.77; *P*<.001), 2 (
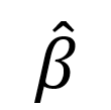
= 0.58, 95% CI 0.32-0.83; *P*<.001), 4 (
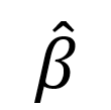
=0.49, 95% CI 0.20-0.78; *P*=.001), and 5 (
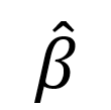
=0.75, 95% CI 0.44-1.08; *P*<.001).

**Table 5:** Adjusted average acceptability ratings (out of 5, where 1=unhelpful and 5=very helpful) for each module.

	Value, mean (SE; 95% CI)
Module 1	4.01 (0.12; 3.77-4.25)
Module 2	4.06 (0.14; 3.79-4.34)
Module 3	3.49 (0.15; 3.19-3.78)
Module 4	3.98 (0.16; 3.67-4.29)
Module 5	4.25 (0.17; 3.91-4.59)

#### Association of Module Completion and Acceptability With Participant Demographics and Distress Levels

No association was detected between module acceptability and education, farm type, remoteness, age, internet use, or baseline psychological distress (Multimedia Appendix 1). Acceptability ratings were related to stress scores (
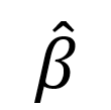
=0.14, 95% CI 0.06-0.22; *P*=.001); the more stressful the events of the past month, the more satisfied participants were with the modules. Acceptability ratings were related to gender at *P*=.08, indicating a possible trend toward females finding the modules more satisfying than males.

No association was detected between the number of modules completed and gender, education level, farm type, remoteness, hours of internet use per week, or baseline psychological distress or stress exposure (Multimedia Appendix 2). There was an association between module completion and age, with older participants completing more modules (
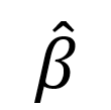
=0.03, 95% CI 0.00-0.06; *P*=.04). Finally, there was no association between module completion and an individual’s average acceptability rating (*r*=−0.04; *P*=.52) or their rating of the last module they completed (*r*=0.10; *P*=.28).

### Stage 8b: Usability and Acceptability Testing of Prototype (Qualitative Data)

#### Brief Phone Calls With Users Who Exited the Intervention Before Completion of All 5 Modules (to Find Out Why)

Table 6 summarizes the most frequently identified reasons for not completing a module (N=108). Most often, farmers said they were too busy or simply had not got to it yet (86/108, 79.6%). The next most common reason was that the content was not relevant to them (14/108, 13%) or that they had forgotten about it (8/108, 7.4%).

**Table 6:** Reasons for not completing all 5 modules (N=108)^a^.

Reason	Number of mentions, n (%)
Too busy or not got to it yet	86 (79.6)
Not relevant or helpful for me	14 (13)
I forgot or thought I had done it	8 (7.4)
Technical issues: user end	7 (6.5)
Module took a while or too long	7 (6.5)
Repetitive questions	7 (6.5)
My health	5 (4.6)
Technical issues: ifarmwell	4 (3.7)
Forgot password or reset issues	2 (1.9)

^a^Some participants gave ≥1 reason.

#### Detailed Phone Interviews With Purposively Sampled Group of Users

In total, 4 broad themes and 25 subthemes were identified and are outlined in Table 7. The themes included the following: using ifarmwell was a positive experience, value for themselves but unsure how best to recommend to others, areas for improvement, and context. The findings generally indicated that users found ifarmwell easy to use and navigate, relevant, credible, and necessary, particularly because of the tough drought conditions that many farmers were experiencing at the time of data collection. Farmers generally liked the structure of the modules and the time provided between modules to practice strategies. They also consistently reported that the language, videos, and cartoons were appropriate, the email or text reminders were helpful, and they valued the opportunity for self-reflection and the anonymity and privacy of the resource. Findings regarding areas for improvement included using even more farmer-focused language, improving the sound quality of the audio files, and including additional reminder SMS text messages to address forgetfulness. Module 3 was also identified as too long, and the values exercises it contained were found to be difficult for people who had never considered this type of value clarification exercise before. The need to double click to answer questions on iPads and iPhones was also something that users said they needed the website to remind them to do.

**Table 7:** Themes and subthemes from interviews with ifarmwell website users.

Themes			Example quote
**Using ifarmwell was a positive experience**			“When I started it off I thought, ‘These guys have been reading my mind or watching me,’ because it seemed very pertinent, very pertinent. But also, just the fact that there’s no shame. I don't have to be ashamed of the fact that I can't help the things I can't help. That’s a very empowering and liberating sort of a thing, so I got that from you.” [female, 56 years, VIC^a^, sheep and/or cattle property]
	Easy to use and navigate	“Very usable, I was really impressed with the usability of it, it was very simple and I am quite computer literate but I can imagine that someone that perhaps wasn’t so computer literate, the layout and the sequential nature of it, was pretty good.” [female, 61 years, VIC, sheep and/or cattle property]	
	Relatable and relevant to farmers	“Yes, if it was just for the ordinary person, which would be, of course, an urban person, it would be very, very different. I'm very grateful it was something focused on farmers because it just - well, it personalised it. It understands what’s going on.” [male, 64 years, WA^b^, grain, sheep and/or cattle farm] “Because you’ve structured it for farmers. We’re very down-to-earth people, and I think some of these other courses weren’t down-to-earth enough. So, your language is being appropriate, your contents are appropriate, illustrations are brilliant.” [female, 66 years, NSW^c^, horticulture, market garden, and/or fruit growing] “But like it definitely - yeah read as something that was relevant from a rural perspective and approachable I guess, didn’t strike me as someone in an office in Sydney telling us how we should be dealing with the issues of rural mental health or whatever. Like it came across as real.” [female, 33 years, QLD^d^, sheep and/or cattle property]	
	Content credible and well-developed	“I’m just trying to think of the—yeah, I think everything—well, there was nothing in there that I feel was irrelevant or inappropriate at any point.” [male, 42 years, NSW, grain, sheep and/or cattle farm] “Yes, I think it’s quite credible. Everything that was written there it was well written, it was easy to understand. I know it said if you need help call Lifeline. There was, that was on there, so yes, it was quite good I thought. Definitely, it looked good. You have obviously spent a lot of work on it. I found it good.” [female, 31 years, VIC, grain, sheep and/or cattle farm]	
	Appropriate language and explanations	“It was good, it was simple. Not too simple that made you feel like, dumb or anything. They didn’t have big words either that you need to look up. So yes, it was quite appropriate I think for the demographic that you’re trying to target.” [female, 31 years, VIC, grain, sheep and/or cattle farm] “Just the way you chose the words, you didn’t make it more complex than it needed to be and you didn’t use technical jargon, it was very simple, everyday language.” [female, 61 years, VIC, sheep and/or cattle farm]	
	Videos were relatable, accessible, good quality, and useful	Again, I thought they (the videos) were really good because they are relatable and they are real. [female, 55, VIC, Dairy farm]	
	Appropriate use of photographs and cartoons	“Yeah, so happy again with those because they really, I think they were chosen well to reflect the environment of the people that you’re hoping to reach. You know, kept things within that framework, so yeah, no, absolutely happy with all of those.” [male, 25 years, WA, grain, sheep and/or cattle farm]	
	Modules were presented in a logical sequence	“I liked the way that it was broken up into different modules so that you were able to look at a section, do the skills and be exposed to some new skills and then have time to consolidate and think about that. For me, that’s a really good way to learn new skills, rather than just looking at something on that and then going ‘oh that was interesting’, it sort of was dribbled out a little bit over a period of time and I found that a really useful format for developing a structure for reflecting on how you deal with life and I think that’s a really useful way for a lot of farmers too.” [female, 61 years, VIC, sheep and/or cattle farm]	
	Valued time to implement strategies between modules	“It was good because it gave you a chance to practice or think about some of the things that you’d discovered, and then—without overloading you, and then you had another follow-up at the next step. I really liked the way that it did that. Like I said, it made it a much more sustainable sort of process.” [male, 36 years, VIC, viticulture]	
	Email, text, or voicemail, exercises, reminders were helpful	“Yeah, so as much as I hate enlisting in something and they keep bugging me all the time, I thought the texts as well as the things were good, particularly when you’ve got a fortnight between stuff. Yeah, I thought that was really good.” [male, 42 years, SA^e^, grain, sheep and/or cattle farm] “I used to be looking forward to when I got the little message on the phone that said, ‘Oi! It’s time for you to start doing that extra module.’ That’s something, I guess, that’s important for you lot. The fact that you contact us means that it seems that we’ve got a relationship or it seems that we matter.” [female, 56 years, VIC, sheep and/or cattle farm]	
	Practical strategies	“It was good....It was quite practical in the way it was presented, the information was presented...Some of the examples that were presented and things like that were something you can easily identify with. It didn't go into too much detail.” [male, 36 years, VIC, viticulture] “Yeah, I thought it was interesting, I quite like it, I liked the practicality of it actually. I think that was probably the biggest selling point. What I would kind of tell people if I were to recommend it would be there’s a lot of practical advice in there, I think that’s missing in a lot of stuff. So yeah, no that was definitely the high point of it.” [male, 25 years, WA, grain, sheep and/or cattle farm]	
	Using ifarmwell facilitated self-reflection	“I think—it took me a long time to identify and realize that I needed to do something with my mental health. It takes a lot to go forward and speak to someone, so being able to go through those modules on your own and identify where you need—you might need some help or even just identifying a few things that you can do for yourself, I think that probably suits farmers or anyone I've ever dealt with at work. I think being able to do something on your own to start with and get a [00:08:38], if this gives you a bit of information, really, to—then if you want to go to someone, you can say, ‘Look, this is what I think I need help with.’ That’s where I really struggled. I didn't know—I didn't really know what to—if I was going to go and talk to someone, I didn't really know what to say. But now I—having gone through those modules, it really highlighted for me.” [male, 36 years, VIC, viticulture]	
	A necessary and timely resource	“I hope it rolls out because to me it’d be a fair loss if it did not keep going—for sure. So I suppose that means that I better swallow my pride and actually tell someone about it.” [male, 42 years, SA, grain, sheep and/or cattle farm] “I think it’s a good program, you’d say, I suppose. It’s probably what we need right now too.” [female, 31 years, VIC, grain, sheep and/or cattle farm] “So no, I hope it doesn’t disappear because I think there is a definite need there.” [female, 33 years, QLD, sheep and/or cattle farm]	
	Appreciated the opportunity to add tools to Toolbox and refer back to summary sheets over time	“I quite liked the way you could put stuff in your Toolbox. You could find those things that were potentially going to work for you and put them somewhere so you can refer to them later or coming back to them.” [male, 36 years, VIC, viticulture]	
	Able to understand strategies and apply them to life	“Just looking at the things I've printed out and stuck on the wall that I thought—be curious. Yes. Always be curious. Always investigate. Put your attention into the here and now. That is—that’s important.” [female, 71 years, NSW, sheep and/or cattle farm] “I think it was good. Sure I got some pointers and some tips from that as to how to get over the long and low periods. I mean these are common factors but of course at times when you are down and out you can’t think of anything. But these few straight from the program sure help and create that awareness that you can do this or you can do that and give it a go. And it sure help, useful help” [female, 55 years, VIC, sheep and/or cattle farm]	
	Appreciated privacy and anonymity	“And like, in this environment, if you want to go to town and go and see a counsellor or a psychologist to say that you happen to live in an area where there is one there, that’s probably only going to be an every-now-and-again type visit, it is very difficult to - like I can’t even make a doctor’s appointment for a script around here with the whole f****** district knowing. Something like that, nobody needs to know. And I know that that actually goes slightly against what we are trying to say is yeah, it is okay to ask for help and it is okay to reach out but sometimes it is actually good to have that first step or offering people resources that doesn’t involve anybody knowing about it necessarily.” [female, 33 years, QLD, sheep and/or cattle farm]	
	Willing to recommend to peers	“Yeah, so I’d definitely be willing to—I reckon it definitely has a space, it fills a need that isn’t really getting addressed so far.” [male, 25 years, WA, grain, sheep and/or cattle farm] “That’s where I'm doing most of my promoting. I say to the girls—not just girls, to all the people, ‘This ifarmwell thing, it was a brilliant idea because this helps. It’s particularly tailored for farmers.’” [female, 56 years, VIC, sheep and/or cattle farm]	
	Value for themselves but unsure about how best to recommend to others	“Yeah I would definitely and I actually thought, while I was going through, there is probably—well, I actually think it would do my partner a lot of good to do it as well, but I haven’t quite worked out how to encourage him to do that. But I definitely would given the right type of circumstances” [female, 33 years, QLD, sheep and/or cattle farm]	
**Areas for improvement**			
	More farmer-focused language	“You didn’t put enough farmers’ language in there.” [male, 65 years, SA, sheep and/or cattle farm]	
	Improve the sound quality of audio files	“Actually one thing that was a bit of a problem was the, when there was meditations that, the girl that was doing the meditations, her voice was quite low and I couldn’t turn it up. So that was a bit of an issue. I could get through with it but it was, that was something that I did notice” [female, 62 years, TAS^f^, horticulture, market garden, and/or fruit growing]	
	Include additional reminders	“Maybe more reminders. I know for me I obviously signed up and I suppose people that do sign up to do these things do have the intent to do it. Like everything, you sort of get emails from here, there and that’s just life these days and that’s just the way it is. But I would appreciate obviously another reminder being like ‘Come on!’” [female, 23 years, NSW, sheep and/or cattle farm]	
	Shorten module 3	“I think that one [module 3] took me the longest time, actually. I did—I think a lot of those things were relevant, and then after a while I dragged and dropped all these things and I began to regret it a little bit, because it took so much time to sort it out and comment on each one. I think that’s what happened, so it was a bit lengthy.” [male, 64 years, WA, grain, sheep and/or cattle farm]	
	Remind users to double tap to select answers on iPads and iPhones	“The only thing- like there was a note about it was that you had to double tap because I did a fair bit of it on my phone and...a couple of times like you would do your multiple choice and I would have to go back because it would say you haven’t answered it. I’m like, ‘Oh, I did answer it.’ But just so obviously hadn’t but there was a note in there telling you what you had to do and that was fine but I would say that was more operator error than internet thing.” [female, 33 years, QLD, sheep and/or cattle farm]	
**Context**			
	Farmers are time poor	“I am thinking about—from it personally but I am also thinking about it in terms of professionally and how I would perhaps recommend something like that to farmers that I am working with as well and I think that the fact that it’s not a very time consuming thing, each module means that you can just do a little bit at a time and you can jump in and out of it, depending on what time requirements you have so the overall structure I thought was terrific from that perspective.” [female, 61 years, VIC, sheep and/or cattle farm]	
	Mental health stigma	“I guess probably a lot of farmers probably baulk when they hear something about mental health, feelings and emotions and that sort of thing” [male, 36 years, VIC, viticulture] “I think it’s a really good idea because it’s—farmers are very proud people. They won't always go and seek help. But this is kind of non-threatening. They don't have to talk to anybody if they don't want to.” [female, 57 years, TAS, grain, sheep and/or cattle farm]	
	Drought	“We can't do anything about the weather. We can't change it. I haven't got any feed.” [female, 71 years, NSW, sheep and/or cattle farm] “And the other things I liked about it was just that you are farmer-orientated, which is totally different to any of the other help—beyondblue, Black Dog, they’re all just for general people but farming situations are particularly unique and your ‘ifarmwell’ tapped in to that—so the idea that drought or cattle prices that you can't influence and, more importantly, succession.” [female, 56 years, VIC, sheep and/or cattle farm]	
	Women are perceived as most likely to use and recommend	“I think, the wives, I reckon the wives would be more likely to be interested in it.” [female, 62 years, TAS, horticulture, market garden, and/or fruit growing]	

^a^VIC: Victoria, Australia.

^b^WA: Western Australia, Australia.

^c^NSW: New South Wales, Australia.

^d^QLD: Queensland, Australia.

^e^SA: South Australia, Australia.

^f^TAS: Tasmania, Australia.

### Stage 9: Iterative Design Improvements

In response to the findings detailed above, several changes were made to the website. To improve clarity and brevity, minor wording changes and reductions in the text were made in all modules. Audio recordings were professionally rerecorded to improve quality. Edits to the text were also made to acknowledge that accessing a GP can be difficult for those in rural areas, that module 1, in particular, was very long because of the pre-evaluation questionnaires (but that subsequent modules would involve less reading and more activities), and that questionnaires were standardized and only included for the purposes of website evaluation (not part of the intervention itself; eg, cognitive fusion). To improve usability, additional reminders were included about the inability to *go back* and the need to double tap responses if using an iPad or iPhone. A *Things to remember when using this website* page was added to emphasize these key messages. The *save and continue* button was also made more prominent. To improve relevance, additional images and rotating banners were included on the home page to reflect the broader range of demographics of users accessing the website. To improve adherence, additional SMS text messaging reminders were added 7 days after registration if module 1 was not completed and 28 days after the preceding module was completed if the next module was not started for modules 2 to 5 (Figure 2). Module 3 was shortened by removing 1 value clarification exercise that gave users feedback on values they may not be living consistently with (based on their answers to a questionnaire; Table 3). The revised module 3 retains an exercise asking users to select values that are very important to them, think about whether these values drive their behavior and decision-making, and how they might plan to live more consistently with these values in the future.

## Discussion

### Principal Findings

This paper outlines the process of integrating evidence from the literature and consumer and expert advice to create a resource that is informed by evidence and perceived as acceptable and relevant by its users. A strength of this intervention development process was the clear, iterative methodology that allowed the integration of different types of knowledge at each step. This involved the synthesis of evidence from prior research and intervention mapping to identify key determinants of behavior change, relevant behavior change and engagement strategies, and the involvement of farmers as co-designers throughout the process to ensure the acceptability of evidence-based strategies. In particular, the farmers’ feedback was used to inform the initial design of the website, amend the prototype before launch, inform the timing of the launch, and update the intervention following acceptability and usability testing of the prototype. At all stages, farmers’ feedback was prioritized and integrated with research evidence and expert opinions. These approaches enabled us to develop a resource that reflects the unique farming culture, is built on evidence-based approaches to mental health and well-being, demonstrates an understanding of the audience for which it was intended, and as detailed in this paper, was found to be acceptable.

More specifically, the acceptability and usability testing of the prototype that included both quantitative and qualitative components and farmers from a variety of Australian states and farm types, found that once people started a module, most completed that module. Approximately 83.6% (117/140) of users starting module 1 completed module 1, and all people who started modules 4 and 5 completed them. Importantly, acceptability with the previous module was not found to predict whether a user went on to complete the next module, which aligns with the qualitative feedback from people who did not complete every module that their main reason for not progressing was *Too busy/not got to it yet*. Overall, 37.1% (52/140) of people who started module 1 completed the entire 5 module intervention. Comparatively, recent studies have shown a wide variation in the rate of adherence and attrition to web-based interventions for mental health between 2% and 83% [72]. Other studies have reported that approximately 75% do not use mHealth apps more than once after installation [73]. Pleasingly, the present intervention was found to be most acceptable to those who needed it most (ie, those who were most highly distressed when they started module 1) rather than those who were most educated. These high levels of acceptability are significant, given the aforementioned reluctance of farmers to seek help [14], engage with resources targeting their mental health [37], and their general perception that existing services are not designed for them [24]. The intervention also aims to help farmers identify when and how to seek professional help and highlights the role of their local GP. In turn, this may prevent the development of severe mental health problems and facilitate access to treatment at an earlier stage, thereby minimizing the intensity of interventions required and reducing both social and treatment costs. Findings from the qualitative interviews with noncompleters (N=108) to find their reasoning for ceasing participation, also met calls for more research to aid the understanding of engagement in web-based interventions [74] and may be used to inform the inclusion of strategies for improvement in future interventions.

The only comparable farmer-focused well-being website is the aforementioned Scottish CCBT *Living Life to the Full*, which includes personalized support emails in addition to computerized modules [23]. That trial found that of those who logged on (N=35), only 5 (14%) completed the 5 core modules, which is much less than the 37.1% (52/140) reported in this study. Bowyer et al [23] noted that rates of attrition in their study with farmers (73.2%) were much higher than those experienced when they tested a very similar intervention with other population groups (26%-27% attrition) [75,76], reinforcing the notion that the farming population is particularly difficult to engage in health and well-being–focused interventions.

Although acceptability with the ifarmwell modules was generally high, along with the interview comments, they did highlight some areas for improvement. Following the acceptability testing reported in this paper, the website was adapted to address any concerns and improve the user experience. Changes included shortening a module, improving the quality of audio recordings, and incorporating additional SMS text message reminders, which demonstrates the value of adaptive design in building a resource that is responsive to user experiences. This aligns with the person-based approach by continuing to incorporate user feedback after live testing of the intervention [34], which is a strength of this work as it allows interventions to be responsive to the needs of the audience while remaining publicly available. The need to ensure that modules are as short as possible (or can be easily stopped and recommenced) is important for other farming-focused intervention developers to keep in mind. Our finding that farmers lack the time to engage in web-based interventions aligns with findings that more than half of the Australian farming population work ≥50 hours per week, compared with just 16% of the rest of the working population [77].

### Limitations

The sample was limited to those farmers who self-selected to take part in the website evaluation and may not be representative of the wider farming community [78]. Another limitation of this research is that it was not clear to many users that the questionnaires used for evaluation purposes were not part of the intervention itself, which may have contributed to the perception of module length and negatively affected user acceptability. A yellow background was used behind the evaluation components; however, in the future, this delineation should be made even clearer, possibly by having users access the questionnaires via a separate window.

### Further Research

We have demonstrated that a co-designed website is usable and acceptable to farmers, and many of the lessons from this research may be applied to the development of future farmer-focused interventions. However, further research is needed to systematically test the effectiveness of this intervention and examine the psychological mechanisms that facilitate changes (or otherwise) in outcomes. In the case of ifarmwell, analyses should specifically examine whether key ACT processes (Table 3) are influenced by the intervention and, if so, how they relate to any changes in distress and well-being outcomes. This would not only inform further refinements to the ifarmwell website but also help progress important gaps in knowledge about psychological mechanisms in the field of ACT [43,51].

### Conclusions

This paper describes the first web-based intervention co-designed with farmers to help them adopt coping strategies to better manage their stress by accepting things beyond their control and living according to their values, regardless of the circumstances they face. Importantly, this paper outlines the value of a co-design approach in facilitating the development of interventions that are centered on evidence-based therapeutic approaches, that also appeal to audiences who are typically reluctant to seek help for mental health problems. It also details a comprehensive, successful website development and acceptability testing process, which may inform the development of future web-based interventions for difficult-to-reach populations.

## Appendix

Multimedia Appendix 1 Linear mixed model assessing the relationship between module number, demographic and distress variables on module acceptability rating.

Multimedia Appendix 2 Univariable and multivariable linear regression models predicting highest number of modules completed.

## Abbreviations

ACT: acceptance and commitment therapy
CCBT: computerized cognitive behaviour therapy
GP: general practitioner
