# Supplementary material for: Combining Farmers’ Preferences With Evidence-Based Strategies to Prevent and Lower Farmers’ Distress: Co-design and Acceptability Testing of ifarmwell
Source: JMIR Hum Factors. 2022 Jan 11;9(1):e27631. doi: 10.2196/27631 (PMC8790695; doi:10.2196/27631)
Supplement: Multimedia Appendix 1 [file humanfactors_v9i1e27631_app1.docx]

Multimedia Appendix 1. *Linear mixed model assessing the relationship between module number, demographic and distress variables on module acceptability rating.*

|  |  |  |
| --- | --- | --- |
|  | $\hat{\boldsymbol{\beta}}$ **[95% CI]** | ***P value*** |
| Module number |  | <.001 |
| 1 | -0.24 [-0.54, 0.06] |  |
| 2 | -0.19 [-0.50, 0.12] |  |
| 3 | -0.76 [-1.08, -0.44] |  |
| 4 | -0.27 [-0.60, 0.06] |  |
| 5 | - |  |
| Age | -0.00 [-0.02, 0.01] | .52 |
| Gender |  | .08 |
| Female | - |  |
| Male | -0.29 [-0.62, 0.03] |  |
| Education level |  | .25 |
| Primary or high school | 0.01 [-0.44. 0.45] |  |
| University degree or diploma | 0.28 [-0.06, 0.61] |  |
| Postgraduate | 0.37 [-0.12, 0.86] |  |
| Trade certificate | - |  |
| Farm type |  | .90 |
| Dairy | 0.02 [-0.46, 0.51] |  |
| Grain, Sheep and/or Cattle | 0.15 [-0.20, 0.50] |  |
| Horticulture, Market Garden, Fruit | -0.03 [-0.58, 0.53] |  |
| Other | -0.07 [-0.63, 0.50] |  |
| Sheep and/or Cattle | - |  |
| Remoteness |  | .61 |
| Cities and Inner regional | 0.02 [-0.41, 0.44] |  |
| Outer Regional | -0.13 [-0.55, 0.28] |  |
| Remote and very remote | - |  |
| Hours of internet use | 0.01 [-0.01, 0.02] | .54 |
| Baseline psychological distress | -0.01 [-0.04, 0.01] | .28 |
| Baseline stress | 0.14 [0.06, 0.22] | .001 |
| Intercept | 3.59 [2.59, 4.59] | <.001 |
