# Supplementary material for: Combining Farmers’ Preferences With Evidence-Based Strategies to Prevent and Lower Farmers’ Distress: Co-design and Acceptability Testing of ifarmwell
Source: JMIR Hum Factors. 2022 Jan 11;9(1):e27631. doi: 10.2196/27631 (PMC8790695; doi:10.2196/27631)
Supplement: Multimedia Appendix 2 [file humanfactors_v9i1e27631_app2.docx]

Multimedia Appendix 2. *Univariable and multivariable linear regression models predicting highest number of modules completed*

|  | **Univariable Models** | | **Multivariable Model ^s^** | |
| --- | --- | --- | --- | --- |
|  | $\hat{\boldsymbol{\beta}}$ **[95% CI]** | ***P value*** | $\hat{\boldsymbol{\beta}}$ **[95% CI]** | ***P value*** |
| Age | 0.03 [-0.00, 0.05] | .07 | 0.03 [0.00, 0.06] | .04 |
| Gender |  | .65 |  | .90 |
| Female | - |  | - |  |
| Male | -0.16 [-0.86, 0.54] |  | 0.05 [-0.76, 0.87] |  |
| Education level |  | .92 |  | .93 |
| Primary or high school | -0.19 [-1.20, 0.83] |  | -0.31 [-1.39, 0.77] |  |
| University degree or diploma | -0.27 [-1.07, 0.53] |  | 0.02 [-0.86, 0.90] |  |
| Postgraduate | -0.09 [-1.27, 1.10] |  | 0.02 [-1.25, 1.30] |  |
| Trade certificate | - |  | - |  |
| Farm type |  | .48 |  | .94 |
| Dairy | -0.45 [-1.58, 0.68] |  | -0.13 [-1.40, 1.15] |  |
| Grain, Sheep and/or Cattle | -0.14 [-0.94, 0.67] |  | 0.11 [-0.76, 0.99] |  |
| Horticulture, Market Garden, Fruit | -0.52 [-1.78, 0.75] |  | 0.06 [-1.38, 1.50] |  |
| Other | -1.01 [-2.19, 0.16] |  | -0.47 [-1.84, 0.90] |  |
| Sheep and/or Cattle | - |  | - |  |
| Remoteness |  | .92 |  | .98 |
| Cities and Inner regional | -0.19 [-1.16, 0.78] |  | -0.00 [-1.07, 1.07] |  |
| Outer Regional | -0.20 [-1.20, 0.80] |  | -0.08 [-1.14, 0.99] |  |
| Remote and very remote | - |  | - |  |
| Hours of internet use | -0.01 [-0.04, 0.02] | .59 | -0.01 [-0.05, 0.03] | .70 |
| K10 baseline score | -0.04 [-0.09, 0.02] | .16 | -0.02 [-0.08, 0.04] | .52 |
| Stress | -0.09 [-0.26, 0.08] | .31 | -0.08 [-0.28, 0.13] | .45 |
